# A Study of Reverse Characteristics of GaN-on-Si Quasi-Vertical PiN Diode with Beveled Sidewall and Fluorine Plasma Treatment

**DOI:** 10.3390/mi15121448

**Published:** 2024-11-29

**Authors:** Fuchun Jia, Qingyuan Chang, Mengdi Li, Yungang Liu, Ziyan Lu, Jifan Zhang, Jinming Lai, Hao Lu, Yang Lu, Bin Hou, Ling Yang, Xiaohua Ma

**Affiliations:** 1 State Key Discipline Laboratory of Wide Band-Gap Semiconductor Technology, School of Microelectronics, Xidian University, Xi’an 710071, China; 2 The 29th Research Institute of China Electronics Technology Group Corporation, Chengdu 610036, China

**Keywords:** gallium nitride, PiN diode, reverse characteristic, beveled sidewall

## Abstract

In this work, we show a high-performance GaN-on-Si quasi-vertical PiN diode based on the combination of beveled sidewall and fluorine plasma treatment (BSFP) by an inductively coupled plasma (ICP) system. The leakage current and breakdown voltage of the diode are systematically studied. Due to the beveled sidewall treated by the fluorine plasma, the diodes achieve an excellent breakdown voltage (V_BR_) of 790 V and a low reverse leakage current. In addition, the GaN-on-Si quasi-vertical PiN diode achieves a low specific on-resistance (R_on,sp_) of 0.51 mΩ·cm^2^ and a high Baliga’s figure of merit (BFOM) of 1.22 GW/cm^2^. The relationship between the total leakage current and the device diameter shows that the sidewall leakage is the main leakage path of the device. Afterwards, the TCAD simulations based on electric field and electric potential reveal that the fluorine plasma treatment is a major factor in suppressing the leakage current and increasing the V_BR_ for a diode with BSFP. This work systematically analyzes the effects of beveled sidewall and fluorine plasma treatment based on the reverse characteristics of the GaN-on-Si quasi-vertical PiN diode and highlights the great potential of the GaN-on-Si PiN diode for various power applications.

## 1. Introduction

Gallium nitride (GaN)-based transistors and diodes are widely adopted in high-frequency, high-power, and high-temperature applications [1,2]. Due to their ability to be used as power devices, both lateral and vertical GaN devices have attracted increased attention. The lateral power devices, including HEMTs and SBD, have been extensively studied and have practical applications [3,4,5,6]. However, it is notable that the lateral structure is unable to fully utilize the advantages of GaN materials. As compared with the lateral devices, the vertical devices are more competitive for the implementation of high-power applications. This is due to the increasing breakdown voltage without enlarging the devices’ area [7,8,9,10,11]. In addition, the vertical devices can withstand higher current and show superior thermal performance [12]. However, the high cost and small diameter of GaN substrates limit the applications of GaN vertical devices. The GaN vertical devices on Si substrates reduce the cost and promote practical applications.

PiN diodes are fundamental devices for performing rectification and being used as power supplies [13,14,15,16]. Recently, based on the high-quality thick drift layer and by using the field plates’ termination, Y. R. Abdul Khadar et al. designed GaN-on-Si PiN vertical diodes with a breakdown voltage of 820 V, an R_on,sp_ of 0.33 mΩ·cm^2^, and a Barriga’s figure of merit of 2.0 GW/cm^2^ [17]. Although the GaN-on-Si PiN diodes in existing works have shown outstanding performance, more techniques should be developed for further optimizing the reverse characteristics of the device. The reverse characteristics are enhanced by minimizing the leakage current and increasing the breakdown voltage. In GaN-on-Si quasi-vertical PiN diodes, the edges of a P-N junction are located on the sidewalls. This can cause electric field crowding, leading to premature breakdown of the device. Therefore, mitigating the peak electric field at the sidewall is an effective method for improving the reverse characteristics of GaN-on-Si quasi-vertical PiN diodes. Previous studies have shown that the fluorine plasma treatment improves the reverse characteristics by depleting the carriers and suppressing the leakage [18,19]. In addition, the beveled sidewalls also reduce the peak of the electric field [20]. However, using the combination of fluorine plasma and the beveled sidewall for treating the GaN-on-Si PiN diodes has not been systematically researched. When the combination of fluorine plasma treatment and the beveled sidewall is employed, further investigation is required to determine which method contributes more significantly to the optimization of reverse characteristics in GaN-on-Si PiN diodes.

In this work, a combination of BSFP is proposed for the GaN-on-Si PiN quasi-vertical diode. In addition, the main leakage path of the GaN-on-Si quasi-vertical diode is also studied. The GaN-on-Si PiN diode with a combination treatment achieves a breakdown voltage of 790 V and a leakage current density of ~10^−2^ A/cm^2^ at 600 V. We achieve an ultra-low R_on,sp_ of 0.51 mΩ·cm^2^, a current density of 1.5 kA/cm^2^ at 4.3 V, and a BFOM of 1.22 GW/cm^2^. The TCAD simulations of the electric field and electric potential are used to study the major contributions of the fluorine plasma treatment and 70° beveled sidewall.

## 2. Experimental Section

**Figure 1:**
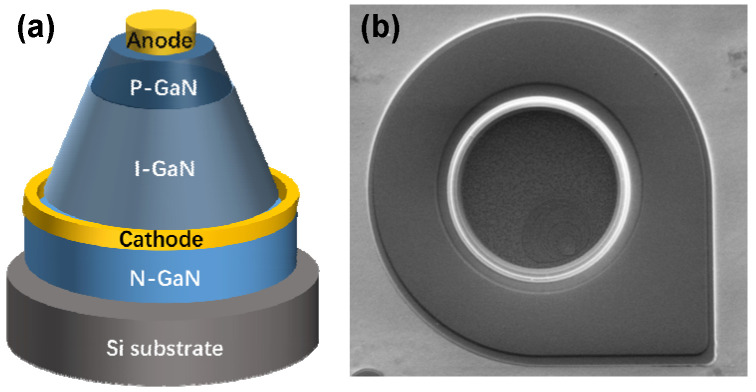
(**a**) The device schematic of the GaN-on-Si quasi-vertical PiN diode. (**b**) Top-view SEM of the GaN-on-Si quasi-vertical PiN diode.

**Figure 2:**
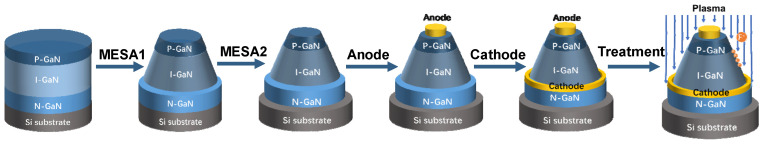
The quasi-vertical diode with BSFP fabrication flow.

Figure 1a,b show the device schematic and top-view SEM of the fabrication flow of the GaN-on-Si quasi-vertical PiN diode used in this work. The proposed material epitaxy structure consists of a 0.4 μm p^+^-GaN layer (N_A_: 3 × 10^17^ cm^−3^), a 4 μm n^-^-GaN layer (N_D_: 2 × 10^16^ cm^−3^), a 1 μm n^+^-GaN layer (N_D_: 1 × 10^19^ cm^−3^), and a high-resistance GaN buffer layer grown on the silicon substrate by using metal–organic chemical vapor deposition (MOCVD). As shown in Figure 2, in order to form a beveled sidewall, the fabrication flow of the GaN-on-Si quasi-vertical diode is started with mesa-1 by using an inductively coupled plasma (ICP) dry etch process. The following process is used to remove the 1 μm n^+^-GaN layer and achieve isolation by using the ICP dry etch. Afterwards, the anode metal, Ni/Au (20 nm/40 nm), is deposited and annealed at 500 °C for 10 min in the presence of O_2_. Then, the main diode structure is finished by depositing the Ti//Al/Ni/Au cathode metal on the top n^+^-GaN layer. In order to achieve the combination of the fluorine plasma and beveled sidewall, the necessary step comprising the CF_4_ plasma treatment by the ICP system for 160 s is applied. During the plasma treatment, the flow of CF_4_ gas is 40 sccm, the ICP coil power is 170 W, and the applied bias power is 35 W.

## 3. Results and Discussion

**Figure 3:**
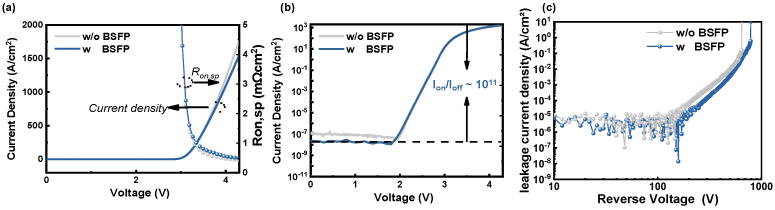
Forward J-V characteristics in (**a**) linear scale and (**b**) semi-log scale for a diode with and without BSFP. (**c**) Reserve J-V characteristics in log scale of devices with and without BSFP.

The forward J-V characteristics of the GaN-on-Si quasi-vertical PiN diode with and without BSFP at room temperature are presented in Figure 3a. During the measurement, the voltage is swept from 0 V to 4.3 V with a step of 0.1 V. The turn-on voltages (V_on_) of the diodes with and without a combination of the fluorine plasma and beveled sidewall are all 3.1 V (extracted at 100 A/cm^2^). The devices with BSFP show a current density of 1.5 kA/cm^2^ at 4.3 V and a R_on,sp_ of 0.51 mΩ·cm^2^. The devices without BSFP show a current density of 1.7 kA/cm^2^ at 4.3 V and a R_on,sp_ of 0.36 mΩ·cm^2^. The devices with BSFP achieve a higher current density and lower R_on,sp_ as compared to the devices without BSFP. Please note that due to the 1 μm thick n^+^-GaN layer, the carrier transport is improved [17]. Therefore, both diodes with and without BSFP achieve a low R_on,sp_. As shown in Figure 3b, the devices with BSFP have a lower off-state leakage current and an on/off ratio (I_on_/I_off_) of 10^11^.

Figure 3c shows reserve J-V characteristics in log scale of the devices with and without BSFP. In this work, V_BR_ is obtained at 1 A/cm^2^. The V_BR_ of devices with and without BSFP is 790 V and 680 V, respectively. The leakage current density of the diode with BSFP is lower than that of the diode without BSFP. The current J of both devices, with and without BSFP, is proportional to V^n^ (n ≈ 7.6 and 6.9, respectively). This demonstrates that the off-state leakage mechanism of the devices in this work is space-charge-limited current (SCLC) [21].

**Figure 4:**
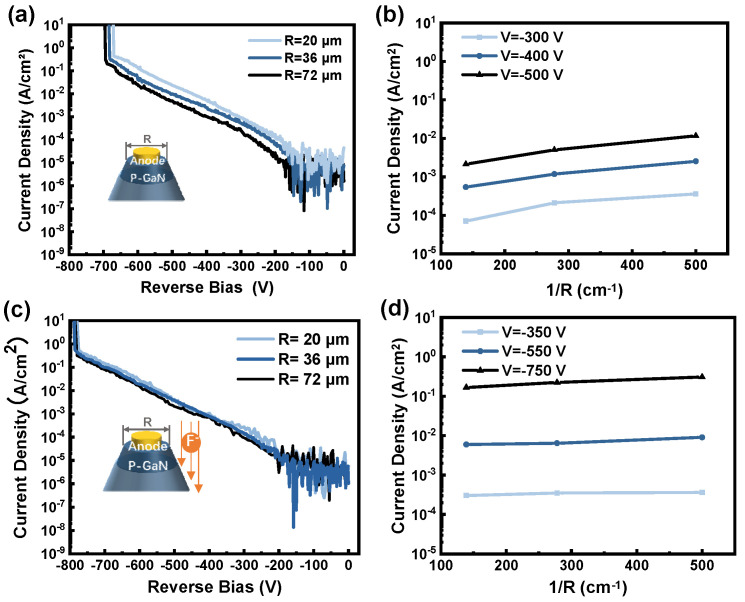
(**a**,**c**) The reverse characteristics of the devices without and with BSFP and with a diameter of 20, 36, and 72 μm. (**b**,**d**) Dependence of the total leakage current on 1/R for didoes without and with BSFP.

In order to clearly analyze the reverse characteristics, the breakdown voltage mechanism and leakage current of devices are further researched. First, the main leakage path of the device is determined by studying the relationship between the total leakage current and the diameter of the device. Figure 4a shows the reverse characteristics of the devices with BSFP and with a diameter of 20, 36, and 72 μm. The total leakage current decreases as the device diameter increases. As shown in Figure 4b, the total leakage current exhibits a linear dependence on 1/R, thus indicating that the sidewall leakage is the main leakage path [22]. As shown in Figure 4c, the total leakage current hardly changes as the diameter of the device decreases. And as shown in Figure 4d, the total leakage current of the devices with BSFP does not change linearly with 1/R. This demonstrates that the sidewall leakage of the devices with a BSFP has been effectively suppressed [22].

**Figure 5:**
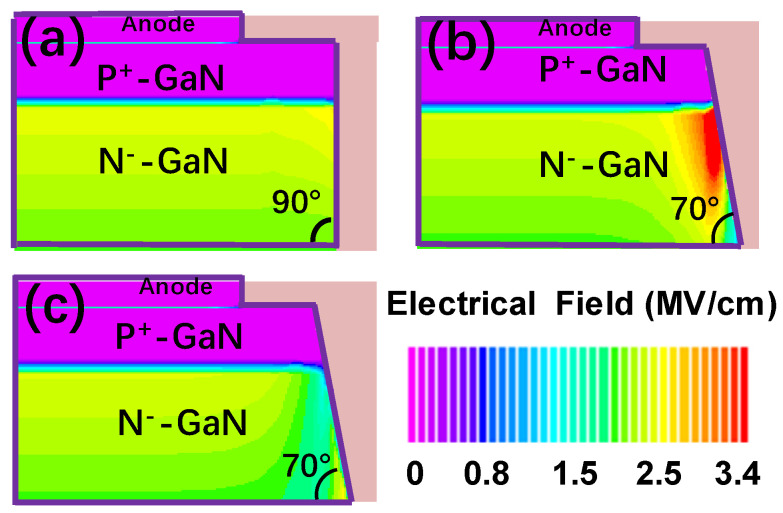
The results of simulated electric field distribution for diodes (**a**) only with fully vertical sidewall, (**b**) only with beveled sidewall, and (**c**) with BSFP at 680 V, respectively.

**Figure 6:**
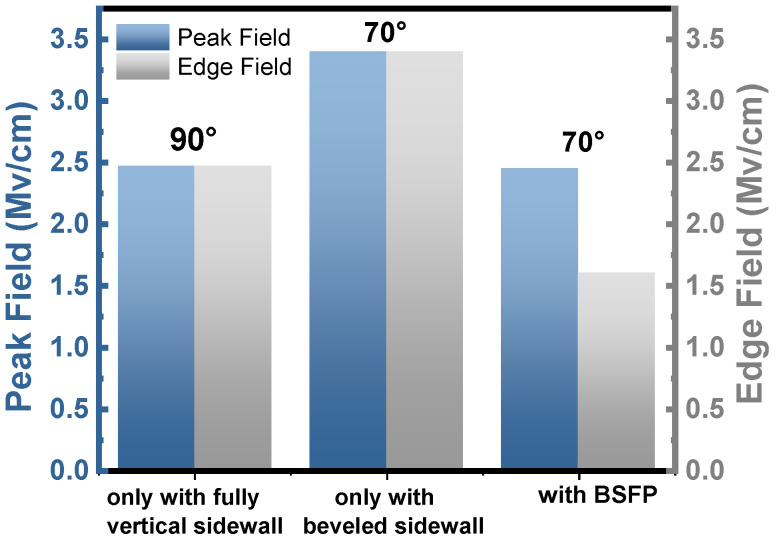
The results of simulated peak field and edge field for diodes only with fully vertical sidewall (90°), only with beveled sidewall (70°), and with BSFP (70°) at 680 V, respectively.

Figure 5a–c show the results of the simulated electric field distribution for the diodes with only a fully vertical sidewall, with only a beveled sidewall, and with BSFP at 680 V, respectively. First, the influence of the beveled sidewall on the reverse characteristics of the device is analyzed based on the simulations. As shown in Figure 5a,b, the results of the TCAD simulation reveal that the fully vertical sidewall mitigates the electric field peak as compared to the beveled sidewall in this work. As shown in Figure 6, as compared to the devices comprising the beveled sidewall only, both the peak field and edge field of the device with a complete vertical sidewall are lower. The beveled sidewall reduces the edge field. However, the key is to keep the angle of the beveled sidewall small enough. In addition, when the angle of the sidewall is less than 20°, the edge electric field of the beveled sidewall device is smaller as compared to the fully vertical (90°) sidewall device [20]. However, as the angle of the beveled sidewall is only 70°, the beveled sidewall in this work does not reduce the electric field.

Now, the influence of fluorine plasma treatments on the reverse characteristics is analyzed. As shown in Figure 5b,c, as compared to the beveled sidewall, the beveled sidewall treated by fluorine plasma effectively mitigates the peak of the electric field. As shown in Figure 6, the edge field of the device with BSFP is lowest. Therefore, the major contribution of mitigating the peak of the electric field is from negative fixed charge formed by the fluorine ions near the surface of the beveled sidewall. In this work, although the 70° beveled sidewall is unable to directly mitigate the peak of the electric field, the influence of the beveled sidewall on the device characteristics is still significant. During the process of self-aligned fluorine plasma treatments, the beveled sidewall easily implants the fluorine ions as compared to the vertical sidewall.

**Figure 7:**
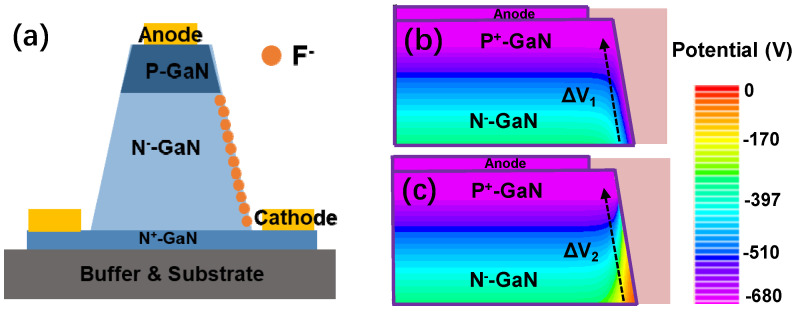
(**a**) The F ion distribution for diode with a BSFP. The simulated potential distribution for diodes (**b**) with and (**c**) without BSFP at 680 V.

Figure 7a shows the location of the fluorine ions in a diode with BSFP. According to prior experimental works presented in the literature, the fluorine ions become negative fixed charges in III-nitride [23,24]. The fluorine ions are mainly concentrated on the surface of the material due to the plasma treatment [25]. Therefore, in this work, the fluorine ions are modeled as negative fixed charges in GaN and are mainly distributed on the surface of the beveled sidewall. Figure 7b,c show the simulated potential distribution of the diodes with and without BSFP at −680 V, where ΔV_1_ and ΔV_2_ denote the changes in the potential near the sidewalls of the diode with and without BSFP, respectively. The simulation results show that ΔV_2_ is greater than ΔV_1_. This means that the potential of the diodes with BSFP changes more slowly as compared to the diodes without BSFP in the region near the sidewall. A slower change in the potential means a lower electric field. Therefore, the electric field near the sidewall of the device with slow potential change is lower. This means that the diodes with BSFP have a lower electric field. In this work, the combination of beveled sidewall and self-aligned fluorine plasma treatment reduces the electric field effectively.

**Figure 8:**
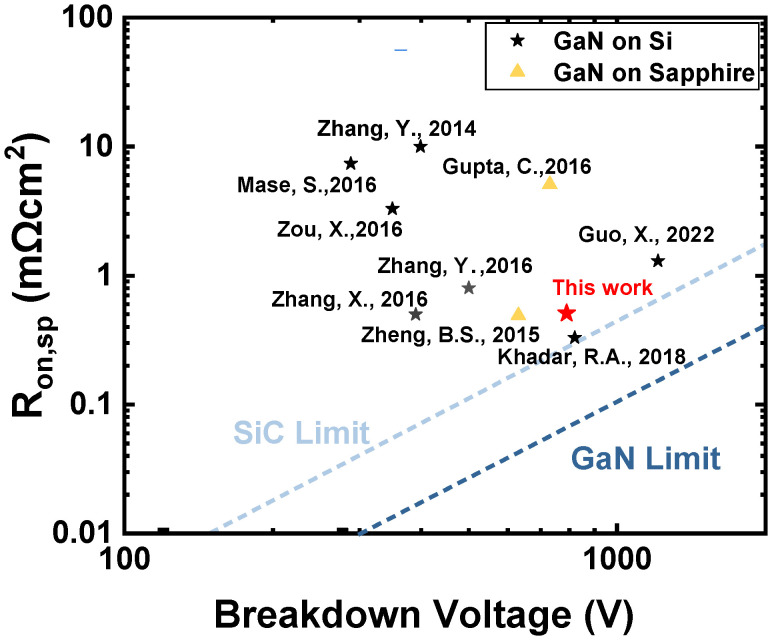
Benchmark of breakdown voltage vs. R_on,sp_ of quasi-vertical PiN diodes with a combination of fluorine plasma and beveled sidewall [9,17,21,26,27,28,29,30,31].

As shown in Figure 8, the performance of the GaN-on-Si quasi-vertical PiN diode in this work is benchmarked against other GaN vertical PiN diodes [9,17,21,26,27,28,29,30,31]. The results show that the PiN diode with BSFP performs better and has a breakdown voltage of 790 V, a low R_on,sp_ of 0.51 mΩ·cm^2^, and a BFOM of 1.22 GW/cm^2^. Table 1 benchmarked the V_BR_, I_ON_/I_OFF_, and I_R_ of the GaN-on-Si quasi-vertical PiN diode in this work against other previously reported GaN quasi-vertical PiN diodes grown on Si substrates. As shown in Table 1, the devices with BSFP have a superior on/off ratio of 10^11^ compared to other GaN-on-Si quasi-vertical PiN diodes [9,17,21,26,27,28,29,30,31]. At the same time, compared with the previous work, the I_R_ (reverse leakage current density) of the diode with the BSFP structure is better suppressed at different reverse bias (200 V and 700 V), showing excellent reverse characteristics.

## 4. Conclusions and Perspectives

In this work, a high-performance GaN-on-Si quasi-vertical PiN diode based on the combination of beveled sidewall and fluorine plasma treatment by the ICP system is fabricated. The reverse characteristics are systematically studied. Due to the combination of fluorine plasma and the beveled sidewall, the diodes achieve an excellent V_BR_ of 790 V and a low reverse leakage current. In addition, the GaN-on-Si quasi-vertical PiN diode achieves a low R_on,sp_ of 0.51 mΩ·cm^2^ and a BFOM of 1.22 GW/cm^2^. The results of leakage analysis show that the beveled sidewall is the main leakage path. The TCAD simulations performed by considering the electric field and electric potential reveal that the fluorine plasma treatment is a major factor in suppressing the leakage current and increasing the V_BR_ for a diode with BSFP. The results demonstrate that the GaN-on-Si vertical power devices have great potential in terms of being used as power transistors and other low-cost applications.

## Figures and Tables

**Table 1:** Benchmark of reverse performance of GaN on Si PiN diode.

Reference	V_BR_ (V)	I_on_/I_off_	I_R_ (200 V) (A/cm^2^)	I_R_ (700) (A/cm^2^)
[21] MIT,14	>300	-	~10^−2^	-
[29] NIT,16	288	~10^10^	10^−4^–10^−3^	-
[27] HKUST,16	390	~10^7^	10^−3^–10^−2^	-
[26] MIT,16	>500	-	10^−4^–10^−3^	-
[17] EPFL,18	820	-	10^−4^–10^−3^	10^0^–10^1^
[31] SINANO,22	1200	~10^10^	10^−7^–10^−6^	10^−3^–10^−2^
This work	790	~10^11^	10^−5^–10^−4^	10^−2^–10^−1^

## Data Availability

The original contributions presented in the study are included in the article, further inquiries can be directed to the corresponding author.
